# The Association Between Psychological Distress and Cancer in Adults: A Community-Based Study in Bogor, Indonesia

**DOI:** 10.34172/jrhs.12763

**Published:** 2026-08-15

**Authors:** Sulistyowati Tuminah, Lely Indrawati, Siti Isfandari, Irmansyah Irmansyah, Dwi Hapsari, Delima Delima, Novia Susianti, Siswanto Siswanto, Laurentia Konadi Mihardja

**Affiliations:** ^1^National Research and Innovation Agency, Research Organization for Health, Research Center for Public Health and Nutrition, Indonesia; ^2^National Research and Innovation Agency, Research Organization for Health, Research Center for Pre-Clinical and Clinical Medicine, Indonesia; ^3^Pulmonology and Respiratory Medicine Department, Universitas Gadjah Mada Academic Hospital, Yogyakarta, Indonesia; ^4^Faculty of Medicine, University of Malahayati, Lampung, Indonesia

**Keywords:** Stress, Psychological, Malignant neoplasm, Tumor, Indonesia

## Abstract

**Introduction:**

Cancer is a leading cause of death, and psychological distress (PD) is one of its potential risk factors. Therefore, this study aimed to assess the association between PD and cancer in adults.

**Study Design:**

A repeated cross-sectional study.

**Methods:**

This study used retrospective data from the non-communicable disease risk factor (NCDRF) community-based study performed in Bogor, Indonesia. All NCDRF and Cohort Study participants meeting the inclusion criteria were included in this study. PD was measured using the Self-Reporting Questionnaire from the World Health Organization, with at least 6 of 20 answered "yes". The study focused on PD in the last 2 years before the first self-reporting cancer diagnosis, and the data sample was analyzed using logistic regression.

**Results:**

Of 2,133 participants, 86 reported a cancer diagnosis, and 278 experienced PD at least once in the last 2 years preceding cancer diagnosis. Compared to those without PD, the risk of cancer diagnosis was significantly higher among individuals who experienced PD, with an odds ratio of 3.67 (95% confidence interval [CI]: 2.143–6.283; P<0.001) for once, and 16.35 (95% CI: 7.663–34.876; P<0.001) for twice, adjusted for age, total cholesterol level, weight loss, and physical activity.

**Conclusion:**

PD was associated with cancer diagnosis. Accordingly, it is important to integrate professional medical advice, psychological motivation, spiritual guidance, and consistent emotional and tangible support from the family to prevent and control PD.

## Introduction

 Psychological distress (PD) is broadly defined as a negative and unpleasant physical or mental situation arising when the stress is too overwhelming or persistent. Distress can be considered a negative psychological state under pressure.^1^ In addition, prolonged distress, or distress that exceeds an organism’s endurance, leads to PD, subsequently inducing a pathological condition.^1^

 According to Kreitler^2^, some studies find the intensity and frequency of reported stress precede cancer diagnoses, indicating that stressful life events (e.g., the loss of a spouse or another family member by death) increase the chance of cancer.^2^

 Cancer is a leading cause of death and a critical barrier to increasing life expectancy in every country.^3^ The cases in the Southeast Asia Region are projected to rapidly rise, increasing from 2.37 million new cases in 2022 to 4.04 million by 2045.^4^ The estimated global economic cost of cancers from 2020 to 2050 is $25.2 trillion in international dollars (at constant 2017 prices).^5^

 Moreover, preclinical studies demonstrate that distress promotes tumorigenesis, tumor progression, and metastasis, and impairs antitumor therapy.^1^ Several clinical studies have shown an inconsistent relationship between PD and cancer. In most cases, observational studies are methodologically (e.g., the design of the studies dealing with psychological factors) inadequate and generally small in size. In addition, there is not enough control of other factors that could have affected the results, and even inverse associations have been reported between distress and cancer.^2,6^

 Therefore, this study seeks to assess the association between PD and cancer. By analyzing individuals who were initially cancer-free and tracking their health for two years before a diagnosis, researchers hope to develop better community-based prevention and management programs in order to reduce the high costs and health risks associated with PD.

## Methods

###  Data Availability

 Data were obtained from the Health Development Policy Agency, Ministry of Health, Republic of Indonesia, with the provisions and procedures that apply through layanandata@kemkes.go.id.

###  Data Source

 A repeated cross-sectional study uses secondary retrospective data from a community-based study of non-communicable disease risk factor (NCDRF), which was performed for six years in Bogor, Indonesia, among citizens aged 25–65 years (in the recruitment process). The respondents’ recruitment was conducted in 2011 and 2012.^7^

###  Participants

*Population:* Participants of the NCDRF Cohort Study in Bogor, Indonesia.

*Inclusion Criteria:* subjects were included in this study if they fully attended every NCDRF Cohort Study activities (three times a year) for six years of monitoring (2011-2017 and 2012-2018) and had no prior cancer diagnosis based on self-reports or a negative result from any examination in the baseline study.

*Exclusion Criteria:* Incomplete data. Nonetheless, The participants who fully attended every NCDRF Cohort Study activities for six years and had no prior cancer diagnosis based on self-reports or a negative result from any examination in the baseline study.

*Minimizing Selection Bias:* The study excluded anyone who already had cancer at the start and only tracked participants who remained in the study for the full six-year duration.

*Minimizing Information Bias:* To ensure that a participant’s mental state was not due to their cancer diagnosis, the researchers only used PD data collected in the last two years before the participants’ first self-report of cancer diagnosis. By using a “disease-free” baseline, the study confirmed that PD preceded the cancer diagnosis.

###  Variables

 The dependent variable (outcome) -collected every two years- is a participant’s first self-reported cancer diagnosis, which is a composite variable derived from cancer-related questions, based on self-reports or examination results indicating malignancy. Due to the low prevalence of individual cancers based on cancer types, “cancer” was used as a general term. Participants with a cancer diagnosis in the baseline study were eliminated, and the cumulative number of self-reported cancer diagnoses that first appeared in the 2nd, 4th, or 6th year of monitoring with binary responses (yes/no) underwent analysis.

 The primary independent variable and the covariate variables were drawn from the two years preceding the appearance of the first self-reported cancer diagnosis. Additionally, the primary independent variable, PD annually, was measured using the 20 questions of the Self-Reporting Questionnaire (SRQ-20) from the World Health Organization, which was translated into the Indonesian language and included “yes/no” answer options. At least six “yes” responses were identified as PD.^8,9^ Based on the two screening results for two years, the experienced PD was categorized into none, once, and twice groups.

 In this study, the reliability of the SRQ-20 was calculated, with a Cronbach’s alpha (∝) of 0.838, which is higher than the “r” table value (0.062), indicating that all the questionnaire items related to PD were reliable.

 Age was classified into under 55 and 55 year-old or above groups. Statistics demonstrated that the peak age range for cancer occurrence was 55–74, with a slow decline after 80 and a low incidence up to 44.^2^ In general, cancer was less common in females than in males.^2^ A family history of cancer data was obtained from respondents’ confessions. In this case, family included the father, mother, siblings, children, grandparents, and other second-degree relatives. Further, a family history of cancer was a composite variable composed of several variables, with yes/no answer options.

 The body weight (BW) is measured three times a year. The percentage of weight loss (WL) is obtained by calculating the difference between the BW measurement at 12 months and 4 months before the cancer diagnosis, compared to the BW measurement at 12 months before the cancer diagnosis. The result is multiplied by 100%. For this variable, there were yes (WL ≥ 2%) and no (others = WL < 2%; BW 12 months ≤ BW 4 months) answers.

 Data on total cholesterol (TC) levels (measured every two years) were obtained from fasting blood lipid levels for 10–12 hours preceding the examination. Ghahremanfard et al^10^ revealed that a lower serum TC level is found in several cancer types. Hence, TC levels were grouped into < 200 mg/dL and ≥ 200 mg/dL categories as the reference category in our study.

 PA data (measured annually) are based on a composite calculation of the type and duration of activity (days per week and minutes per day), including the sport performed. Subjects are categorized as inadequate PA (less than 600 metabolic equivalents for tasks and adequate PA (600 metabolic equivalents for tasks or more) in one week.^11^

 Smoking status (determined annually) is grouped into non-smoking (never smoking and not passive smoking), passive-smoking (never smoking and passive smoking; used to smoke occasionally and passive smoking; used to smoke every day and passive smoking), and active-smoking (currently smoke every day or currently smoke occasionally) categories. Unfortunately, there are no data for passive smoking in subjects who actively smoke.

###  Statistical Analysis

 Participant characteristics were compared using chi-square tests (*P*< 0.05; Table 1). Simple and multiple logistic regressions were used to determine crude odds ratios (crude OR; Table 2) for bivariable and AORs (Table 2) for multivariable analysis, respectively. Moreover, statistical significance was set at *P*< 0.05, and 95% confidence intervals were considered for both bivariable and multivariable analyses. The obtained data were statistically analyzed using IBM SPSS, version 24.0 (IBM Corporation). Eventually, the confounders were evaluated and determined using a cutoff of 10% or more for the difference between the OR full model and reduced model.

**Table 1:** Proportion of Self-Reported Cancer Diagnosis Based on Participants’ Characteristics (N = 2,133)

**Variables**	**With Cancer**		**Without Cancer**		
	**Number**	**Percentage**	**Number**	**Percentage**	***P*** **-Value**
Psychological distress in the last 2 years					
None Once Twice	51	2.7	1,804	97.3	0.000
	22	9.5	209	90.5	
	13	27.7	34	72.3	
Age (year)					
< 55 ≥ 55	15	2.3	642	97.7	0.009
	71	4.8	1,405	95.2	
Gender					
Female Male	60	3.6	1,548	96.4	0.130
	26	5.3	463	94.7	
Family history of cancer					
No Yes	79	4.1	1,857	95.9	0.866
	7	3.6	190	96.4	
Total cholesterol level (mg/dL)					
≥ 200 < 200	31	2.8	1,081	97.2	0.003
	55	5.4	966	94.6	
Weight loss (≥ 2%)					
No Yes	67	3.6	1,790	96.4	0.016
	19	6.9	257	93.1	
Physical activity					
Adequate Inadequate	36	2.4	1,492	97.6	0.000
	50	8.3	555	91.7	
/Smoking status					
Not smoking Passive smoking Active smoking	19	2.4	757	97.6	0.003
	37	4.2	847	95.8	
	30	6.3	443	93.7	

**Table 2:** Bivariable and Multivariable Analyses of the Association Between Psychological Distress and Self-Reported Cancer Diagnosis According to Participants’ Characteristics (N = 2,133)

**Variables**	**Crude OR (95% CI)**	***P*** **-Value**	**Adjusted OR (95% CI)**	***P*** **-Value**
Psychological distress in the last 2 years				
None Once Twice	1.00		1.00	
	3.72 (2.21, 6.26)	0.000	3.67 (2.14, 6.28)	0.000
	13.53 (6.74, 27.16)	0.000	16.35 (7.66, 34.88)	0.000
Age (years)				
Under 55 55 or above	1.00		1.00	
	2.16 (1.23, 3.80)	0.007	2.60 (1.44, 4.69)	0.001
Gender				
Female Male	1.00		1.00	
	1.48 (0.93, 2.38)	0.102	Not retained	
Family history of cancer				
No Yes	1.00		1.00	
	0.87 (0.39, 1.90)	0.720	Not retained	
Total cholesterol level (mg/dL)				
200 or above Under 200	1.00		1.00	
	1.99 (1.27, 3.11)	0.003	1.71 (1.07, 2.74)	0.026
Weight loss (≥ 2%)				
No Yes	1.00		1.00	
	1.98 (1.17, 3.34)	0.011	2.15 (1.23, 3.75)	0.007
Physical activity				
Adequate Inadequate	1.00		1.00	
	3.73 (2.41, 5.79)	0.000	4.30 (2.70, 6.84)	0.000
Smoking status				
Not smoking Passive smoking Active smoking	1.00		1.00	
	1.74 (0.99, 3.05)	0.053	Not retained	
	2.70 (1.50, 4.85)	0.001	Not retained	

*Note*. CI: Confidence interval; OR: Odds ratio.

## Results

 Overall, 2,272 individuals who fully attended the activities (three times a year) for 6 years of monitoring were selected from the 5,312 participants of the NCDRF Cohort Study. Then, 2,258 cases who had complete data on PD and cancer diagnosis were chosen of whom, 2,212 cases not having a cancer diagnosis at the baseline study were selected for inclusion.

 Next, 14 participants’ data with missing on baseline BW were excluded from the investigation. Considering that there are no extreme fluctuations in BW data over time, the average of before and after measurements was inputted if the BW data were missing during monitoring. Additionally, 65 participants’ data with missing on lipid examinations (2 from the baseline study, 40 from 2 years of monitoring, and 23 from 4 years of monitoring) were excluded, and finally, data from 2,133 participants were analyzed in this research (Figure 1).

**Figure 1:**
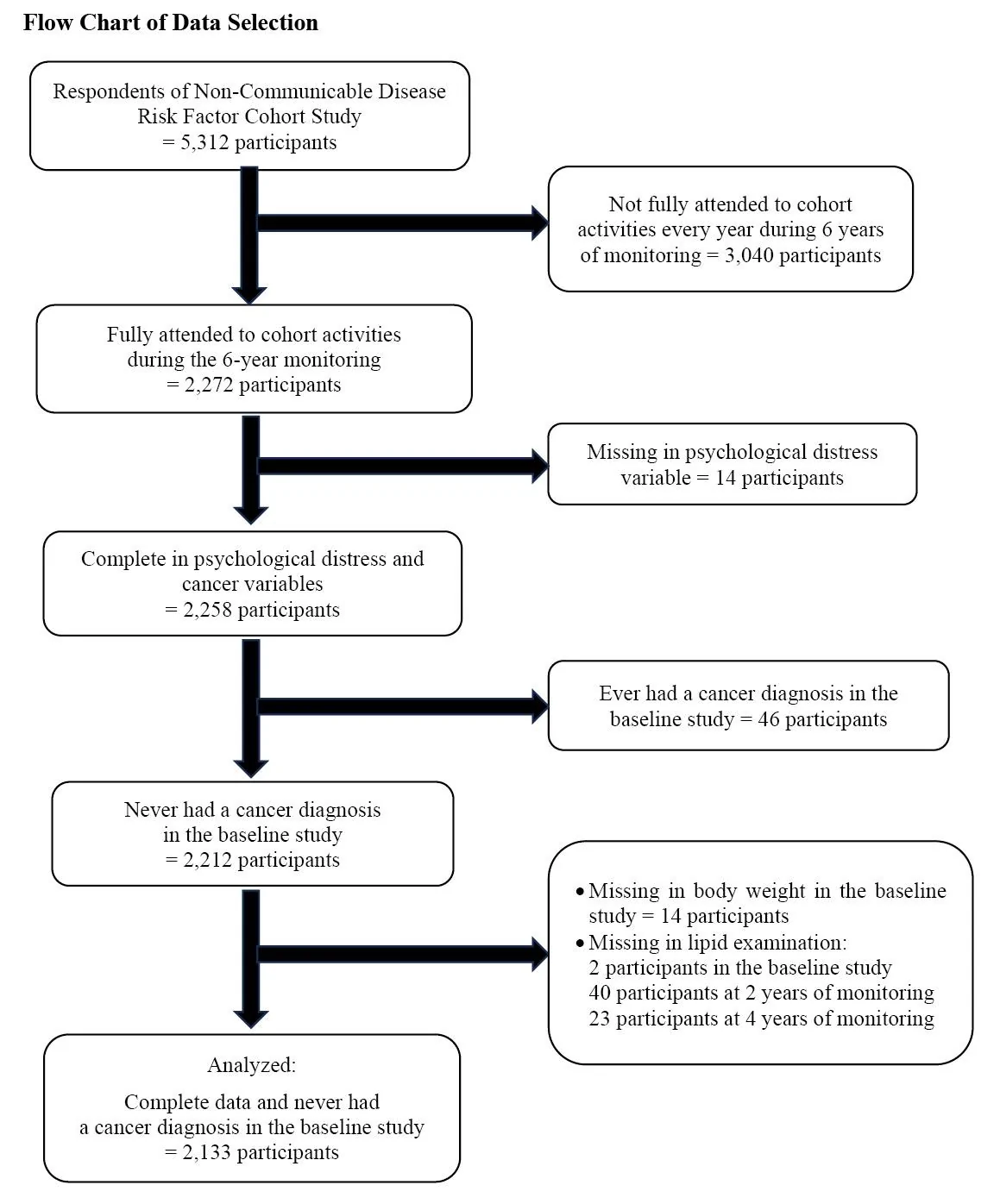
Flow Chart of Sample Selection for Analysis of the Association Between Psychological Distress and Self-Reported Cancer Diagnosis

 Our findings revealed that of the 2,133 participants in the NCDRF Cohort Study, about 4.0% (86 participants) received a cancer diagnosis during the 6-year monitoring period, 10.8% experienced PD once (231 participants), and 2.2% (47 participants) experienced it twice in the two years preceding their cancer diagnosis. Likewise, the results demonstrated a higher proportion of participants who were aged 55 years or above, were female, had no family history of cancer, had higher TC levels (≥ 200 mg/dL), engaged in adequate PA, exposed to passive smoking, and did not experience WL ≥ 2% (Figure 2).

**Figure 2:**
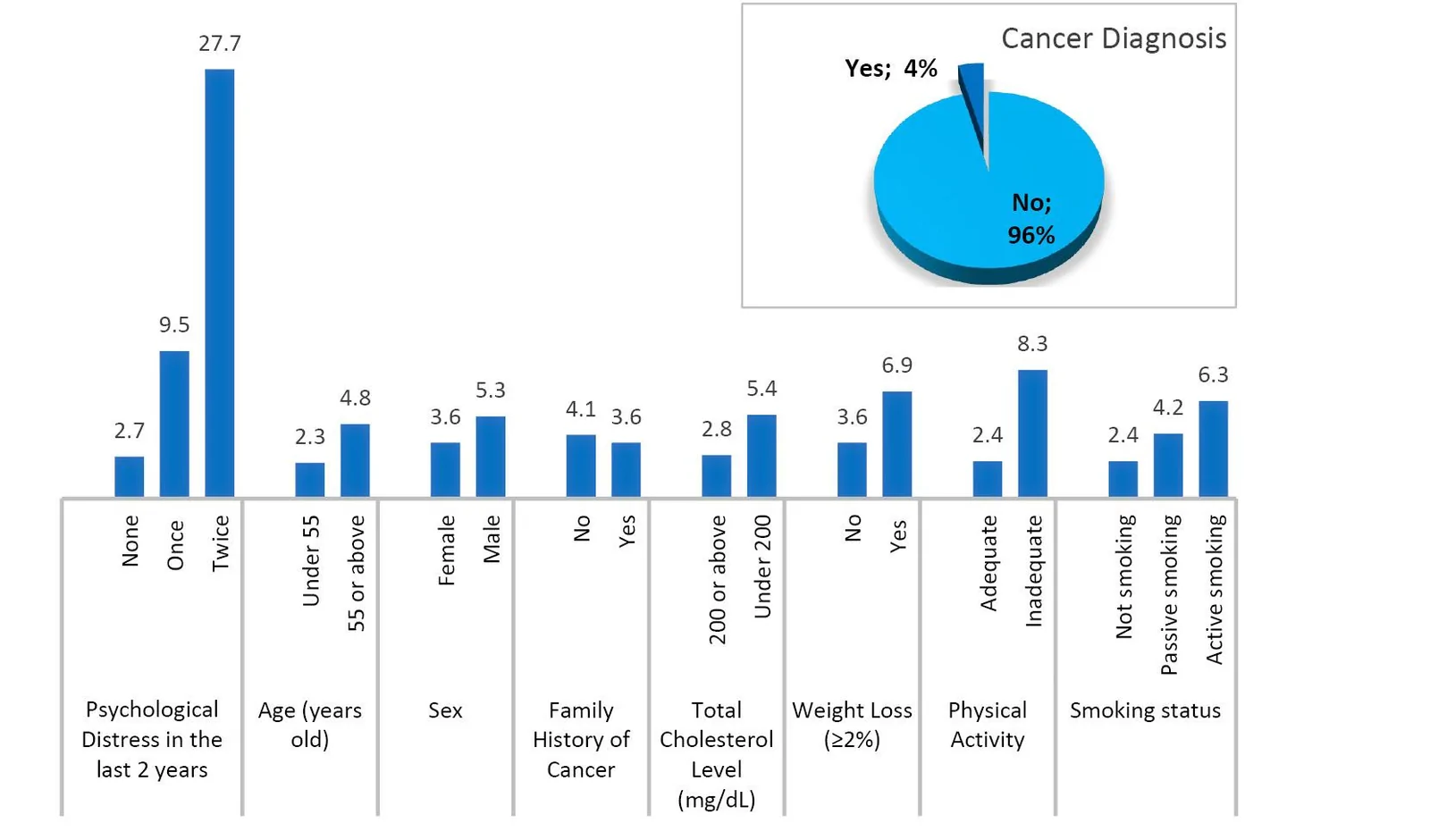
Prevalence of self-Reported Cancer Diagnosis (Top-Right) and Proportion of Participants Based on the Characteristics (N = 2,133)

 Our crosstabulation results (Table 1) indicated a higher proportion of cancer diagnosis among participants who experienced PD twice in the last two years (*P*< 0.001), were aged 55 years or older, had lower TC levels, experienced WL ≥ 2%, had inadequate PA, and were active smoking. Conversely, there were no statistically significant differences in cancer diagnoses based on gender or family history of cancer.

 Our bivariable analysis (Table 2) confirmed that the risk of cancer increased in participants with PD by a 3.72-fold (95% CI: 2.214–6.263; *P*< 0.001) for those who experienced PD once and a 13.53-fold (95% CI: 6.736–27.156; *P*< 0.001) for those who experienced PD twice, which is higher rather than the ones without PD in the last two years.

 The findings represented an increase in the risk of cancer. Participants aged 55 years or above faced a 2.16-times higher risk than those under 55 (95% CI: 1.230–3.804; *P*= 0.007). In addition, participants with a lower TC level (under 200 mg/dL) displayed a 1.99-fold higher risk of cancer compared to those with higher TC levels (95% CI: 1.268–3.110; *P*= 0.003). Furthermore, participants who experienced WL ≥ 2% showed a 1.98-times higher risk than those without WL ≥ 2% (95% CI: 1.167–3.342; *P*= 0.011). Based on the results, participants with inadequate PA had a striking 3.73-fold higher risk of cancer in comparison to those with adequate PA (95% CI: 2.406–5.794; *P*< 0.001), and active smokers had a 2.70-times higher risk compared to non-smokers (95% CI: 1.501–4.850; *P*= 0.001).

 Conversely, gender, family history of cancer, and passive smoking demonstrated no statistically significant increase in cancer risk. For gender and family history of cancer, the ORs were less than one with *P*> 0.05, while for passive smoking, the corresponding ratio was greater than one but still had a significance level of *P*> 0.05.

 Multivariable analysis (Table 2) revealed that a history of PD is associated with 3.67-fold and 16.35-fold increases in the risk of cancer diagnosis for those who experienced PD once (95% CI: 2.143–6.283; *P*< 0.001) and twice (95% CI: 7.663–34.876; *P*< 0.001), which is higher rather than that of those without PD in the last 2 years after adjustments for age, TC levels, WL ≥ 2%, and PA. Even though the variable of PD with the category “twice” in the last 2 years seemed to have a higher association with cancer diagnosis, the precision was lower because the cases were small. Smoking status in this analysis underwent a change, where active smoking in the bivariate analysis was statistically significantly associated with cancer diagnosis. Unfortunately, the association was attenuated after adjusting for the covariates. It was found that physical activity (PA) becomes the most influential factor in the association between PD and cancer. Ultimately, our results confirmed that a lower TC level influences this association as well.

## Discussion

 Cancer develops over many years from exposure to a causative agent to subsequent clinical manifestation and diagnosis.^12^ Nadler and Zurbenko^13^ reported that 35 of 44 cancer types develop at least 10 years before cancer diagnosis, with wide variability across cancer types.^13^ It is challenging to determine when the real incidence of cancer occur and what real risk factors cause cancer initiation in humans. People are frequently unaware of an abnormal biological initiation until symptoms (i.e., unusual changes/growths in their body or organ function) cause discomfort. Nicholson et al^14^ concluded that people seek cancer treatment and get a diagnosis when it is already in an advanced stage.

 Our findings demonstrated that PD was associated with cancer diagnosis, especially when it occurred at least once in two measurements in the last two years before the diagnosis, which is consistent with Kreitler’s book^2^, revealing that the presence of traumatic grief symptoms about 6 months after the spousal loss (e.g., prolonged emotional distress) predicted the diagnosis of cancer after the 13–25-month follow-up. It further suggests that the risk consists not in the stress of bereavement as such but in the emotional and mental health.^2^ This may be due to the PD they experienced, a higher intensity or heavier symptoms based on the SRQ-20, or longer duration, even though the duration is less than a year.

 Cooper et al^15^ noted that the impact of stress on cancer risk varies by the type, frequency, duration, and timing of stressful events. Acute stressors have a slight impact, while chronic stressors (e.g., caregiving or traumatic events) create a greater stress burden.^15,16^ Chronic stress, characterized by prolonged intensity and duration, can suppress immune function through the dysregulation of the hypothalamic-pituitary-adrenal axis and the sympathetic nervous system.^17^ In addition, immune suppression may impair surveillance against oncogenic processes, facilitating tumor growth and metastasis.^18^ The cumulative burden may interfere with cellular repair processes, facilitate chronic inflammation, and speed up carcinogenesis.^19^ Moreover, stress-related hormones can influence tumour development, migration, invasion, and cancer cell proliferation.^20^

 Our findings regarding a significantly higher cancer risk for those aged 55 and older align with global data. Statistics show that the peak age range for cancer occurrence is 55–74 years, with a slow decline after age 80 and a low incidence up to age 44.^2^ Similarly, our results are in line with those of Wang et al^21^, representing that serious PD was positively associated with cancer in adults (50 years or more).

 Our study found that lower TC levels (< 200 mg/dL) were a significant factor in the association between PD and self-reported cancer diagnosis, which conforms to the findings of Ghahremanfard et al^10^, indicating that lower TC levels in breast, colorectal, gastric, and ovarian cancers ranged between 170.1 ± 35.0 and 196.3 ± 43.4 mg/dL. Our results also support the findings of the study by Kitahara et al^22^, showing that the overall incidence of cancer decreased as TC levels increased in both men and women.

 It is believed that cancer cells tolerate excessive metabolic consequences of plasma cholesterol intake to sustain cancer progression, which may explain why the observed serum cholesterol levels in some cancer patients were lower.^23^ Low cholesterol concentration is also considered a potential biomarker for cancer since several types of cancer appear to decrease plasma cholesterol levels. Increasing cholesterol utilization by the tumors accelerates their development and increases their aggressiveness.^24^

 However, some studies reported that the relationship between cholesterol and cancer risk varies by cancer type.Ding et al^23^ found that higher cholesterol was linked to increased risk of breast, prostate, and colorectal cancers while not being related to bladder or lung cancer.

 Most cancers associated with unexpected WL are diagnosed at a late stage. Nonetheless, there was also evidence for an association between unexpected WL and the diagnosis of stages II and III cancers.^14^ Wang et al^25^ revealed that WL > 10% of BW was related to a significantly higher incidence of cancer within a year than was no WL. The data in this manuscript showed no self-reported cancer cases when using a WL threshold of ≥ 10%. To prevent empty data cells, a lower 2% WL threshold was adopted instead.

 Our findings indicated that inadequate PA is the most significant and influential factor linking PD to cancer diagnosis, which matches the results of research suggesting that PD often triggers detrimental behavioral changes, such as reduced PA, poor diet, and increased smoking, which all elevate cancer risk.^6^ Inadequate PA significantly increases cancer risk by suppressing immune function, promoting chronic inflammation, and creating a tumor-friendly microenvironment.^26^

 PAs were performed in Indonesia in 2017 through the Healthy Community Movement; however, it faced challenges due to low public awareness and insufficient local government infrastructure.^27^

 Regarding mental health, community screening using SRQ-20 in Indonesia began in 2024 with a recommended frequency of at least once per year. These screenings are set to become a mandatory component of Indonesia’s universal health coverage.

 The study’s internal validity was bolstered by minimizing selection and information bias and evaluating potential confounding variables. However, this study had certain limitations. The biannual collection of cancer history may have missed diagnoses occurring between intervals. In addition, the study design did not allow for a causal analysis of PD or a specific breakdown of cancer types and stages.

 To effectively prevent and control mental health issues, a robust support network is essential. Such a network must integrate professional medical advice, psychological motivation, spiritual guidance, and consistent emotional and tangible support from the family. It is noteworthy that healthcare professionals should not only advocate for social support, but also specifically advocate for support from an internal family member. By strengthening the support system in the family, it is possible to provide effective interactions that guarantee family members’ psychological well-being.

**Highlights**

- Psychological distress in the last two years was associated with cancer diagnosis.
- Lower total cholesterol and weight loss ≥ 2% were the new findings in this association.
- A support network was found to be essential for preventing and controlling mental health problems.
- This network encompassed medical, psychological, spiritual, and emotional support.

## Conclusion

 Our findings revealed that PD at least once in the last two years is positively correlated with a self-reported cancer diagnosis, after adjusting for age, TC level, WL, and PA.

## Acknowledgments

 The authors would like to thank all parties who have helped in the process of creating this scientific paper, the technical staff from the Data Management Laboratory of the Center for Data and Information, namely, the Ministry of Health of the Republic of Indonesia as the data provider, and other individuals who facilitated the implementation of this research.

## Artificial Intelligence Use Disclosure

 The abstract and main text were refined using proofreading tools, such as Grammarly and Google Gemini (https://gemini.google.com/app?hl=id), to improve grammatical accuracy.

## Competing Interests

 The authors have no conflict of interests associated with the material presented in this paper.

## Ethical Approval

 This study received financial approval from the Health Research Ethics Commission, National Institute of Health Research and Development (KEPK-BPPK), Ministry of Health, Republic of Indonesia (No. LB.02.01/2/KE.108/2017 dated March 27^th^, 2017, and No. LB.02.01/2/KE.076/2018 dated March 1^st^, 2018). The data collection process began with a briefing on the research activities to the participants. Each participant then signed a voluntary informed consent form. Other non-authorized parties are not permitted to use the participant’s identity.

## Funding

 The authors received no financial support for this article’s research, authorship, and publication.
